# Characterization of Non-O157 *Escherichia coli* from Cattle Faecal Samples in the North-West Province of South Africa

**DOI:** 10.3390/microorganisms7080272

**Published:** 2019-08-20

**Authors:** Emmanuel W. Bumunang, Tim A. McAllister, Rahat Zaheer, Rodrigo Ortega Polo, Kim Stanford, Robin King, Yan D. Niu, Collins N. Ateba

**Affiliations:** 1 Department of Microbiology, Faculty of Natural and Agricultural Sciences, North-West University, Mafikeng Campus, Private Bag X2046, Mmabatho 2735, South Africa; 2 Agriculture and Agri-Food Canada, Lethbridge Research and Development Centre, Lethbridge, AB T1J 4B1, Canada; 3 Alberta Agriculture and Forestry, Lethbridge, AB T1J 4V6, Canada; 4 Alberta Agriculture and Forestry, Edmonton, AB T6H 4P2, Canada; 5 Department of Ecosystem and Public Health, Faculty of Veterinary Medicine, University of Calgary, Calgary, AB T2N 1N4, Canada

**Keywords:** non-O157 *Escherichia coli*, cattle, antibiotic resistance, biofilm, virulence genes, PFGE, WGS

## Abstract

*Escherichia coli* are commensal bacteria in the gastrointestinal tract of mammals, but some strains have acquired Shiga-toxins and can cause enterohemorrhagic diarrhoea and kidney failure in humans. Shiga-toxigenic *E. coli* (STEC) strains such as *E. coli* O157:H7 and some non-O157 strains also contain other virulence traits, some of which contribute to their ability to form biofilms. This study characterized non-O157 *E. coli* from South African cattle faecal samples for their virulence potential, antimicrobial resistance (AMR), biofilm-forming ability, and genetic relatedness using culture-based methods, pulsed-field gel electrophoresis (PFGE), and whole genome sequencing (WGS). Of 80 isolates screened, 77.5% (62/80) possessed Shiga-toxins genes. Of 18 antimicrobials tested, phenotypic resistance was detected against seven antimicrobials. Resistance ranged from 1.3% (1/80) for ampicillin-sulbactam to 20% (16/80) for tetracycline. Antimicrobial resistance genes were infrequently detected except for *tetA*, which was found in 31.3% (25/80) and *tetB* detected in 11.3% (9/80) of isolates. Eight biofilm-forming associated genes were detected in STEC isolates (n = 62) and two non-STEC strains. Prevalence of biofilm genes ranged from 31.3% (20/64) for *ehaA^β^* passenger to 100% for curli structural subunit (*csgA*) and curli regulators (*csgA* and *crl*). Of the 64 STEC and multi-drug resistant isolates, 70.3% (45/64) and 37.5% (24/64) formed strong biofilms on polystyrene at 22 and 37 °C, respectively. Of 59 isolates screened by PFGE, 37 showed unique patterns and the remaining isolates were grouped into five clusters with a ≥90% relatedness. In silico serotyping following WGS on a subset of 24 non-O157 STEC isolates predicted 20 serotypes comprising three novel serotypes, indicating their diversity as potential pathogens. These findings show that North West South African cattle harbour genetically diverse, virulent, antimicrobial-resistant and biofilm-forming non-O157 *E. coli*. Biofilm-forming ability may increase the likelihood of persistence of these pathogens in the environment and facilitate their dissemination, increasing the risk of cross contamination or establishment of infections in hosts.

## 1. Introduction

Shiga-toxigenic *E. coli* (STEC) strains such as *E. coli* O157 and non-O157 (e.g., O26, O45, O91, O103, O104, O111, O113, O121, O118, O128, O145, O148 and O174) have acquired genetic traits that make them pathogenic to humans. These strains have caused both sporadic illness and outbreaks of food and water-borne infections worldwide [1,2,3]. Infections range from simple diarrhoea to the more complicated haemorrhagic colitis (HC), haemolytic uremic syndrome (HUS) and thrombotic thrombocytopenic purpura [4]. The consumption of contaminated food and water is generally known to be the most common mode of transmission [5]. Beef and dairy cattle are considered the main reservoir of these pathogens [6]. Oral rehydration is the principal method for the treatment of symptoms such as diarrhoea linked with *E. coli* infections [7]. The use of antibiotics for *E. coli* infections, especially STEC, remains a cause for concern as some strains exhibit resistance to a variety of antimicrobials, and antimicrobial therapy can heighten toxin production in STEC, increasing the risk of HUS [4,8]. *E. coli* strains that produce Shiga toxins (Stx) are termed Shiga-toxigenic STECand possess variants of the *stx1* and *stx2* genes, respectively [9]. Despite the fact that Shiga-toxins are the main virulence factors of STEC [10], additional accessory virulence factors such as adhesins, pili, intimin and hemolysin also contribute to pathogenicity.

A biofilm is the aggregation of microbial cells on a surface surrounded by a protective extracellular polymeric matrix [11]. Biofilms are major contributors to the persistence of undesirable bacteria in both food-processing plants and hospitals, as they are tenacious and resist disinfection or treatment [12,13]. This situation is exacerbated by the fact that most STEC has a very low infectious dose (i.e., <10 cells) [14]. Mature biofilms occasionally rupture, dispersing daughter cells [15], which can form new biofilms on contact surfaces or food [16,17,18]. In nature, bacteria predominantly exist within biofilms [19] and these communities are also frequently associated with infections in humans [20]. It has also been reported that non-O157 STEC forms biofilms on food contact surfaces. Therefore, biofilms could serve as potential reservoirs for food contamination, spoilage and sources of infection for consumers [21].

Attention has been directed towards the detection and characterization of *E. coli* O157:H7 in South Africa [22,23,24] and particularly in the North West Region [25,26]. However, previous studies investigating non-O157 STEC are limited. This study characterized non-O157 *E. coli* from cattle faecal samples for their virulence potential, antimicrobial resistance, biofilm-forming ability and genetic relatedness in consideration of the potential for disease associated with non-O157 STEC strains.

## 2. Materials and Methods

### 2.1. Sample Collection and Bacteria Isolation

A total of 600 faecal samples were collected from February 2015 to March 2017. Collection was performed from 3 commercial beef and/or dairy farms in 3 regions (Rooigrond, Vryburg and Koster) of the North-West Province (Mafikeng) of South Africa. Samples were transported on ice and analyzed immediately upon arrival in the laboratory. One gram of faecal sample was suspended in 7 mL MacConkey broth medium (Biolab Merck, Gauteng, South Africa) and incubated aerobically at 37 °C for 4 h. After incubation, 10-fold serial dilutions were prepared in sterile, distilled water and 100 μL aliquots of each dilution were spread-plated onto sorbitol-MacConkey agar (Sigma-Aldrich, St. Louis, MO, USA). Plates were incubated aerobically at 37 °C for 24 h. Presumptive non-O157 *E. coli* isolates (pink) were sub-cultured onto sorbitol MacConkey agar and the plates were incubated aerobically at 37 °C for 24 h. The sub-cultured bacteria were preserved in 50% (*v*/*v*) glycerol and stored at −80 °C for further microbiological studies (Figure S1 in the Supplemental Material). Genomic DNA was extracted from overnight bacterial cultures prepared in Luria-Bertani broth (Merck, Darmstadt, Germany), using the ZR Fungal/Bacterial DNA MiniPrep^TM^ kit (Epigenetics Company, Irvine, CA, USA) according to the manufacturer’s instructions. Using PCR primers targeting *E. coli uidA*, 450 isolates were confirmed as *E. coli* [27]. PCR reactions were prepared in 25 μL total volumes comprised of 1 μM of the template DNA, 50 pmol of each oligonucleotide primer set, DreamTaq Green PCR Master Mix (2 X) (Thermo Scientific, Toronto, ON, Canada) and nuclease-free water. Thermal cycling conditions using a C1000 Touch^TM^ Thermal Cycler (Bio-Rad, Hercules, CA, USA) were as follow: 95 °C for 5 min, followed by 35 cycles of 95 °C for 30 sec, 59 °C for 30 sec, 72 °C for 1.5 min and a final extension at 72 °C for 10 min. Positive *E*. *coli uidA* isolates were subsequently run in an *E. coli* O157 specific PCR [28] to eliminate *E. coli* O157 isolates from further analyses. PCR conditions for *rfbO157* detection were as follows: 95 °C for 3 min, 10 cycles of 95 °C for 1 min, 65 °C for 2 min, 72 °C for 90 s at 72 °C for 10 min. Eighty of the presumptive non-O157 *E. coli* isolates were randomly selected from different regions (Rooigrond dairy n = 22, Rooigrond beef n = 28, Koster dairy n = 20 and Vryburg beef n = 10) and transported as bacterial glycerol stocks to the Lethbridge Research and Development Centre, Canada in accordance with Public Health Agency of Canada Regulations (https://www.canada.ca/en/public-health/services/laboratory-biosafety-biosecurity/human-pathogens-toxins-act.html, http://www.tc.gc.ca/eng/tdg/page-1296.html).

### 2.2. PCR Based Detection of Virulence Genes

Genomic DNA was extracted from the overnight bacterial culture in LB broth (Merck, Germany), using the NucleoSpin^®^ Tissue Kit (Macherey-Nagel, Bethlehem, PA, USA). The purity and concentration of the DNA were determined using the Nanodrop Lite spectrophotometer (Thermo Fisher Scientific, Verona, WI, USA). Bacterial isolates were screened for AMR [29] and biofilm-forming genes by PCR (Table S1 in the Supplemental Material). PCR reactions were performed in a 25 µL volume comprised of HotStar Plus MasterMix (Qiagen, Mississauga, ON, USA), 1 μM of the template DNA, 0.2 μM of each primer set and nuclease-free water. Multiplex PCR was used for the detection of the virulence genes [30]. The PCR mixture comprised QuantiFast Master Mix (Qiagen), 1 μM of the template DNA, 0.2 μM of each primer set and nuclease-free water. *E. coli* O157:H7 strain R508 and *E. coli* O26 strain (EC19960464), which carry *stx2*, *hlyA*, *eaeA* and *stx1* genes, respectively, which were used as positive controls. Amplicons were resolved by gel electrophoresis using 2% (*w*/*v*) agarose at 70 V for 1 h, stained with gelRed and visualized using a UV transilluminator Gel Doc (BioRAD, Hercules, CA, USA). In each gel, a 1 kb plus molecular marker (Thermo Scientific), negative and positive controls were also included.

### 2.3. Antimicrobial Susceptibility Assay 

Antimicrobial resistance (AMR) was determined on 80 non-O157 *E. coli* isolates using the disc diffusion technique [31]. Eighteen antibiotics (BD, Mississauga, ON, Canada) were tested: Ampicillin-sulbactam (AMS; 10/10 µg), amoxicillin-clavulanate (AMC; 20/10 µg), ampicillin (AMP; 10 µg), aztreonam (AZT; 30 µg), cefoxitin (FOX; 30 µg), cefotaxime (FOT; 30 µg), ceftazidime (TAZ; 30 µg), cefepime (CPM; 30 µg), imipenem (IPM; 10 µg), meropenem (MRP; 10 µg), gentamicin (GEN; 10 µg), streptomycin (STR; 10 µg), tetracycline (TET; 30 µg), colistin (CL; 10 µg), chloramphenicol (CHL; 30 µg), nalidixic acid (NAL; 30 µg), norfloxacin (NOR; 10 µg) and trimethoprim-sulfamethoxazole (SXT; 1.25/23.75 µg). The discs were placed on inoculated Mueller-Hinton agar (Dalynn Biologicals, Calgary, AB, Canada) and incubated aerobically at 37 °C for 18 h. Zones of growth inhibition were measured using a Biomic automated zone reader (Giles Scientific, Santa Barbara, CA, USA). Reference values [31] were used to classify isolates as resistant, intermediate resistant and susceptible to a particular antimicrobial agent (Table S2 in the Supplemental Material). *E. coli* ATCC 25922, *Pseudomonas aeruginosa* ATCC 27853 and *E. coli* ATCC 35218 were used as standards as described in Clinical and Laboratory Standards Institute (CLSI) guidelines [31].

### 2.4. Biofilm Formation Assay

Based on virulence gene and AMR profiles, 62 *stx* positive and 2 multidrug-resistant (non-STEC) isolates, were selected for biofilm evaluation in 96-well polystyrene microtiter plates (Nunc, Edmonton, AB, Canada) using a modification of methods described by Wang et al. [17]. Bacterial strains were grown overnight at 37 °C in minimal salt (M9) medium (Sigma-Aldrich) supplemented with 0.4% glucose, 0.02% MgSO_4_·7H_2_O and 0.001% CaCl_2_ (*w*/*v*) without casamino acid for 24 h. After incubation, the cultures were diluted (1:10) in M9 broth and 200 µL of diluted cultures were transferred into the wells in 8 replicates. Plates were incubated at 22 °C or 37 °C for 24 h, 48 h or 72 h. M9 broth only without bacteria was used as a negative control. After incubation, planktonic cells were removed, and wells were washed thrice with sterile water to remove the remaining unattached cells. Plates were fixed with 250 µL of absolute methanol (analytical grade, >99%, Sigma-Aldrich) per well for 20 min at room temperature. After being drained and air-dried, the biofilms were stained with 0.5% (*w*/*v*) crystal violet (Sigma-Aldrich) solution for 20 min. Plates were then washed 3 times with water and air-dried at room temperature. Crystal violet bound to the biofilm was then dissolved by adding 200 µL of 33% glacial acetic acid (Sigma-Aldrich) per well. An aliquot of 125 µL of 33% glacial acetic acid solution was removed from each well, transferred to new microplates and the optical density (OD_590 nm_) was measured in an ELISA plate reader (Synergy^TM^ HT BioTek). The optical density cut-off value (ODc) of 0.082 was determined to be 3 standard deviations above the mean OD of the negative controls. According to Stepanović et al. [32], strains were classified as follows: OD ≤ ODc, non-adherent; ODc < OD ≤2 X ODc, weakly adherent; 2 X ODc < OD ≤4 X ODc, moderately adherent and 4 X ODc < OD strongly adherent based on OD_590 nm_ values obtained as a result of biofilm formation.

### 2.5. Pulsed-Field Gel Electrophoresis (PFGE)

Non-O157 *E. coli* isolates were typed using a Clamped Homogeneous Electric Field-Dynamic Regulation (CHEFDRIII) system (Bio-Rad, Hercules, CA, USA) as described by Ribot et al. [33]. Briefly, agarose-embedded DNA of non-O157 *E. coli* was digested for 2 h with 20 units of *XbaI* restriction enzyme (New England Biolabs, Pickering, ON, Canada). Restriction fragments of DNA were separated on a 1% SeaKem Gold (SKG) agarose (Lonza, USA). The electrophoresis conditions for non-O157 *E. coli* were based on the Centre for Disease and Control (CDC) [34] protocol comprising an initial switch time of 6.76 s, final switch time of 35.38 s, voltage 6 V, angle: 120° and a run time of 18 h. Gel images were captured on a Gel Doc imaging system (Alpha Innotech, San Leandro, CA, USA), and analyzed with BioNumerics software version 7.6 (Applied Maths, Sint-Martens-Latem, Belgium). *Salmonella* Braenderup reference standard (H9812) was used as a control and for standardization of the gels. Band similarity was calculated by applying the dice coefficient method with an optimization of 0.5% and a band matching tolerance of 1%. Cluster analysis was performed using the unweighted pair group methods arithmetic average algorithm to construct a dendrogram. 

### 2.6. Whole Genome Sequencing (WGS)

Of the 80 isolates investigated in this study, 24 isolates representing different sampling regions, virulence and AMR gene profiles, biofilm-forming ability on polystyrene and PFGE profiles were selected for WGS. WGS was performed at the Agri-Food Laboratories, (Alberta Agriculture and Forestry, Edmonton, AB, USA). DNA was quality checked and quantified using a Qubit fluorometer (ThermoFisher, Waltham, MA, USA) and a Tapestation 4200 system (Agilent, Santa Clara, CA, USA). Sample libraries were prepared using the Nextera XT library preparation kit protocol (Illumina, Inc., San Diego, CA, USA). Sequencing was performed on the Illumina MiSeq platform using the MiSeq Reagent Kit V3 to produce 251 bp paired-end reads. Sequencing reads were de novo assembled into contigs using the Shovill pipeline (https://github.com/tseemann/shovill). Shovill included trimming, which was performed with Trimmomatic 0.38, and de novo assembly was performed with SPAdes version 3.13.0. [35]. Draft genome assemblies were annotated with Prokka [36]. Contigs were searched against databases for serotype determinants, virulence factor genes, AMR genes and plasmids using ABRicate version 0.8.7 (https://github.com/tseemann/ABRICATE). Non-O157 *E. coli* serotype determinants (O- and H-antigen sequences) were inferred in silico using the EcOH database (https://doi.org/10.4225/49/571996 C105 E03), which was originally developed to work with the Short Read Sequence Typing for Bacterial Pathogens (SRST2) program [37]. The EcOH database contained sequences of the O-antigen loci [either *wzx* (O-antigen flippase) and *wzy* (O-antigen polymerase)], or the ABC transporter (*wzm* and *wzt*)) and H-antigen (*fliC* and *flnA*) with referenced loci in the *E. coli* O-groups and H-types. The virulence factor (VF) profile was generated by searching contigs against the *E. coli*_VF database (https://github.com/phac-nml/ecoli_vf). Virulence factors were considered as present if the nucleotide sequence identity was above 70% compared to reference gene length. Antimicrobial resistance gene profiles were generated by searching contigs against the Comprehensive Antibiotic Resistance Database [38], and plasmid search profiles were generated by searching contigs against the replicon sequences from the plasmidFinder database (https://cge.cbs.dtu.dk/services/PlasmidFinder/). Replicon sequence identity above 80% was considered present against each genome.

### 2.7. Statistical Analyses

Biofilm results compiled from 3 replicates were scored as weak biofilm (non and weakly adherent) and strong biofilm (intermediate and strongly adherent) based on the criteria of [32]. The optical density of the biofilm was log transformed prior to analysis using the mixed model procedure of SAS (SAS 9.4, SAS Institute, Cary, NC, USA), with isolate * temperature * time as the experimental unit. For mixed model analyses, incubation temperature (22 °C or 37 °C), duration of incubation, and isolate were fixed effects, with replicate treated as a repeated measure. The influence of the source of the isolate and type of farm on presence/absence of genes for AMR was determined by generalized linear mixed models (Proc Glimmix) using a binomial distribution. Model adjusted means (back transformed to original scale) and 95% confidence intervals were reported. The isolate was the experimental unit, with the geographical region of origin and type of cattle (beef or dairy) as fixed effects. The relationships between biofilm formation and biofilm-forming genes; AMR phenotype and AMR genes were assessed using the logistic procedure with Firth’s bias adjustment. For all statistical tests, significance was *p* <0.05.

## 3. Results

### 3.1. PCR Detection of Shiga Toxins and Virulence

The majority of non-O157 *E. coli* strains (75%, 60/80) possessed *stx2*, 65% (52/80) possessed enterohaemolysin A (*hlyA*), while *stx1* and *eaeA* were detected in a small proportion of isolates, 25% (20/80) and 15% (16/80), respectively (Table 1). Twenty percent (16/80) of the isolates harboured both *stx2* and *stx1* genes, while the Shiga-toxin *Stx2* a was present in 13.8% (11/80) of isolates (Table 1). However, it was noted that PCR detection of Shiga toxins and virulence genes was not consistent after the second sub-culturing of the same bacterial glycerol stock (Figure S1 in the Supplemental Material).

### 3.2. Antimicrobial Susceptibility of Non-O157 E. coli

Susceptibility to 18 antimicrobials was tested, with resistance observed for eight antimicrobials (AMS; β-lactam/β-lactamase inhibitor combinations, AMP; Ampicillins, STR; Aminoglycosides, CHL; Phenicols, TET; Tetracyclines, NAL; Quinolones, NOR; Fluoroquinolones and SXT; Folate), representing eight different drug classes (Table 2). All strains were pan-susceptible to AZT, FOX, FOT, TAZ, CPM, IPM, MRP, GEN and CL. Four isolates presented intermediate-resistance to AMC. The antimicrobials to which resistance was most commonly detected were tetracycline and streptomycin. The presence of resistance genes for tetracycline (*tetA* and *tetB*), chloramphenicol (*catA1*), streptomycin (*aadA1*) and beta-lactams (*blaTEM-1*) was assessed (Table 2). All strains that were ampicillin-resistant contained the *blaTEM-1* gene (*p* < 0.001). Isolates that were AMS-resistant and AMC intermediate-resistant were 151 and 115.6 times more likely to be positive for the *blaTEM-1* gene, respectively, (*p* < 0.01). Three isolates showed phenotypic resistance to chloramphenicol, but *catA1* was not detected. No significant effect of the presence of the *aadA1* gene was observed for streptomycin resistance. A strong association was observed between the presence of *tetA* and *tetB* genes and tetracycline resistance (*p* < 0.05), and tetracycline-resistant isolates were 38.8 (*p* < 0.01) and 425.9 times (*p* < 0.001) more likely to have *tetA* and *tetB* genes, respectively. Overall, the geographic origin of the isolates only affected the prevalence of *tetA* which was greater (*p* < 0.01) in Rooigrond than Koster. Multidrug-resistance (MDR) was only observed in 11 dairy/beef isolates collected from the Rooigrond region, with resistance ranging from two to seven antimicrobials (Table 3).

### 3.3. Biofilm Formation and Detection of Associated Genes

**Figure 1:**
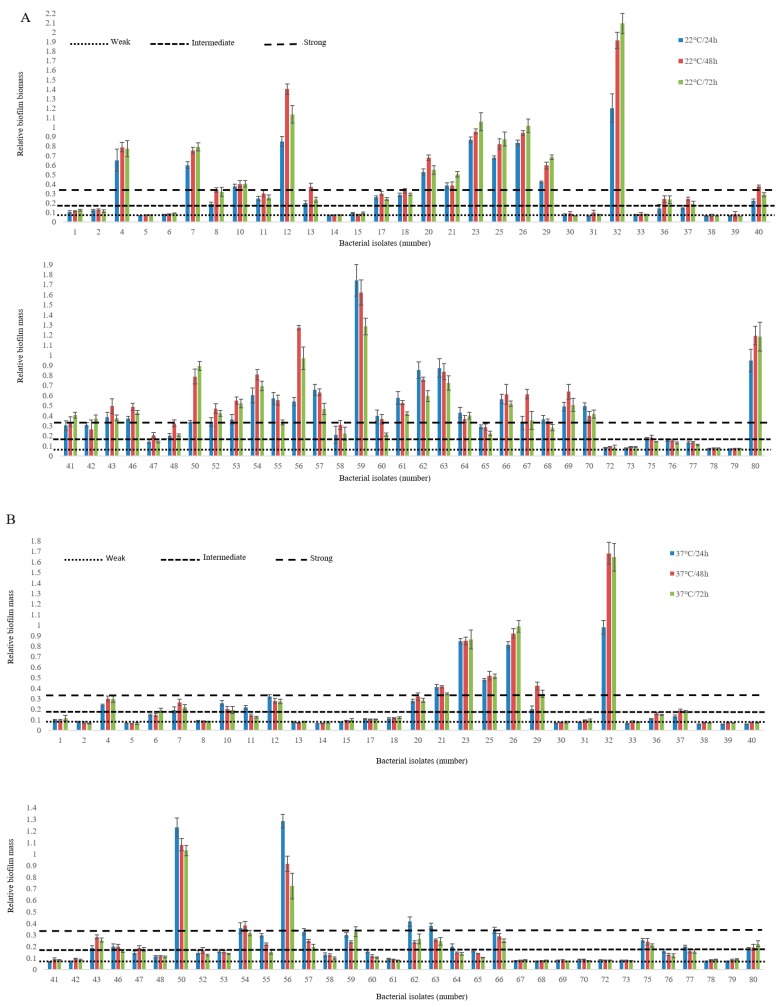
Biofilm formation by non-O157 STEC on a polystyrene surface at 22 °C (**A**) and 37 °C (**B**) using M9 medium. The vertical axis represents the median OD 590 nm of eight replicates of each strain. Horizontal lines represent the cut-off values between weak, intermediate and strong biofilm producers. The OD is defined as three standard deviations above the mean OD of the negative control. Strains were classified as follows: OD ≤ ODc (0.082), non-adherent; ODc < OD ≤ 2 X ODc, weakly adherent; 2 X ODc < OD ≤ 4 X ODc, moderately adherent; 4 X ODc < OD strongly adherent. OD, optical density; ODc, cut-off OD value.

Non-O157 *E. coli* biofilm-forming ability varied amongst isolates, depending on incubation time and incubation temperatures (*p* < 0.001; Figure 1). In most instances, intermediate (OD 0.16) to strong biofilms (OD 0.33) were more likely at 22 °C than 37 °C (*p* < 0.001) and after 48 h (*p* < 0.001) as compared to 24 h and 72 h of incubation (Figure 1). Biofilm-forming ability on polystyrene was strongest at 22 °C and 37 °C in 70.3% and 37.5% of isolates, respectively (Table 4). Biofilm-associated genes including curli structural subunit (*csgA*), curli regulator D (*csgA*), and curli regulator (*crl*) were detected in all isolates. The majority of isolates also carried type 1 fimbriae (*fimH*, 95.3%), DNA binding protein for regulating csgD (*rpoS*, 82.8%), antigen 43 autotransporter protein (*flu*, 76.5%), and Eha passenger (*ehaA^α^*, 60.9%). A lower percentage of isolates were positive for Eha translocation domain (*ehaA^β^*, 31.2%; Table 4). Of the biofilm-forming genes studied, *rpoS* was the only significant predictor of biofilm phenotype at 22 °C (*p* < 0.001) and 37 °C (*p* < 0.01). Overall, biofilm formation log OD at 22 °C and 37 °C were related (*p* <0.001), although the OD at 37 °C only predicted 39% of the variation in OD associated with biofilm formation at 22 °C.

### 3.4. Pulsed-Field Gel Electrophoresis

**Figure 2:**
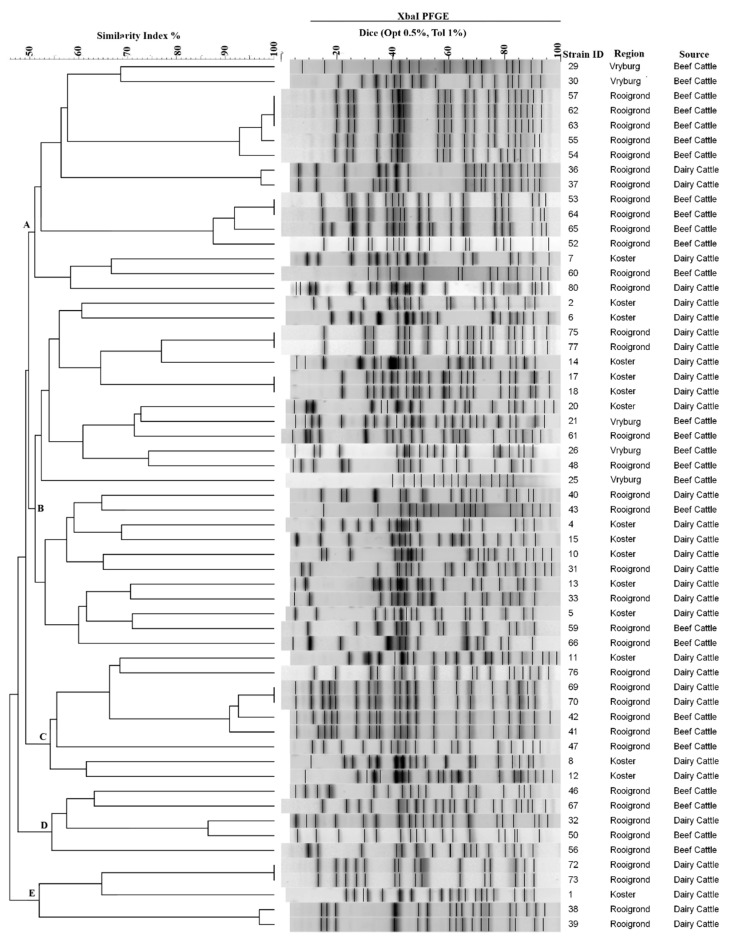
Pulsed-field gel electrophoresis dendrogram of *E. coli* non-O157, strains isolated from cattle (beef and dairy) faecal samples from different locations in the North-West Province of South Africa. *E. coli* genomic DNA was digested with XbaI and the dendrogram was constructed using an unweighted pair-group method.

The *Xba1* digestion of genomic DNA yielded between 14 and 20 bands with sizes ranging from 20.5 and 668.9 kb. Of 64 isolates profiled, five were undigested by *XbaI* and repeatedly appeared as smears. Fifty-nine isolates generated a total of 37 distinct restriction patterns using ≥90% similarity of the Dice coefficient, indicating a high level of diversity amongst bacterial strains from different sampling regions (Figure 2).

### 3.5. Whole Genome Sequence Analyses

Twenty-four isolates examined by WGS in this study had read coverage between 135 and 235 X, with genome sizes ranging from 4,564,407 to 5,562,801 bp (Table S3 in the Supplemental Material). These isolates were classified into twenty different serotypes (Table 5), including three O26:H11 and three isolates with novel serotypes. Annotation of Shiga-toxin genes did not always concur with *stx* detection via PCR (Table 5). Pathogenicity of bacterial strains was characterized based on virulence-associated gene profiling (Table S4 in the Supplemental Material), including: Autotransporter proteins; (*EhaA* and *EhaB*); [39], (*EpeA*); [40], (*Agn43*); [41], adhesins (*Paa* and *EaeA*); [42], (*Ecp*); [43], (*ToxB*); [44], (*CsgA*); [45], invasins (*AslA*, *IbeB* and *OmpA*); [46], (*FimH*); [47], iron uptake (*ChuA*); [48], serine protease (*EspP*); [49], toxins (*HlyA*); [50], (*Cdt*); [51], (*East1*); [52], (*SubA*); [53] and Type III translocated protein (*EspA*); [54].

Of a total of 20 virulence factors (VF) predicted, at least four were present in all the 24 isolates The most common VFs included genes coding for autotransporter protein (*ehaA* and *ehaB*), serine protease (*espP*), toxins (*hlyA* and *suba*), type III translocated (*espA*), invasins (*fimH* and *ibeB*) and adhesins (*ecpA* and *csgA*). Isolates, 11 (O156:H25), 37 (O17:H18), 60 (O116:H11), 64 and 65 (wzx-Onovel5:H19), and 69 and 42 (O26:H11) had the most common VF (Table S4 in the Supplemental Material).

A total of 16 antimicrobial resistance related genes were predicted in the 24 isolates. The most common encoded resistance to polymyxin (*ugd*,) streptogramin (*vgac*), and sulfonamide (*sul2*), which were present in 79.2, 16.6 and 16.6% of isolates, respectively. Interestingly, isolate 56 (O154:H10) carried 10/16 (62.5%) of antimicrobial resistance genes targeted. Six isolates indicated the presence of more than one resistance gene and two isolates harboured resistance to beta-lactams (Table S5 in the Supplemental Material). The presence of resistance genes corresponded with phenotypic resistance based on disc diffusion assay for beta-lactams, streptomycin and tetracycline most especially for isolate 56 (O154:H10).

The 24 non-O157 *E. coli* isolates contained three colicinogenic (Col) and 13 major incompatibility (Inc) plasmids. Seventeen isolates had more than one plasmid. The most frequent plasmid replicons were *IncFIB* (AP001918) and *ColRNAI*, in 62.5% and 50% of isolates, respectively. (Table S6 in the Supplemental Material).

## 4. Discussion

This study is the first to systemically characterize diverse groups of potentially pathogenic non-O157 *E. coli* from cattle faecal samples in the North-West region, South Africa. Non-O157 *E. coli* are emerging pathotypes around the world [5]. Most STEC human infections are attributed to contamination with cattle faeces [55], with STEC-associated disease widely documented in developing countries [7]. Humans may acquire STEC as a result of contamination of meat during processing, or surface and ground waters that are used to irrigate produce or other crops destined for human consumption [56] 

### 4.1. PCR Detection of Shiga-Toxins and Virulence Genes

Non-O157 *E. coli* that possess a high proportion of *stx2* to *stx1* genes as documented in this study have been identified in other studies from South Africa [57], Iran [58] and the United States [59]. However, this observation is contrary to the studies conducted in Western Canada [60] and Europe [61], where non-O157 isolates have been found to harbour more *stx1*- than *stx2* genes. Geographical location, seasonal changes and the presence of Shiga toxin-encoding prophages [62] may contribute to differences in the presence of Shiga toxin genes. Stx2 is regarded as the principal virulence factor in STEC [2] and is more often associated with HUS than Stx1 [63,64]. This study also detected stx2a, a subtype that has been reported to be more virulent than others [65]. Frequent detection of *stx2* in South African non-O157 *E. coli* isolates with subtype *stx2* could be cause for concern as *stx2* has been reported to be more toxic in humans than *stx1* [66]. Surprisingly, in this study, detection of *stx1* and *stx2* via conventional PCR did not conform to the annotated sequences derived by WGS. In this case, 66.7% (14/21) of the isolates that possessed either *stx1* or *stx2* based on PCR were not confirmed to still harbour these genes after WGS. There a number of possible explanations for this observation. Firstly, isolates may have lost the Shiga toxin related genes as a result of repetitive sub-culturing between assessment with PCR vs. WGS. Karch et al. [67] reported that after repetitive culture in liquid and solid media, non-O157 *E. coli* (O2:H5, O26:H11, O73:34 and O100:H32) became negative for either *stx1* or *stx2* gene by PCR and Vero cell assay. Similarly, Joris et al. [68] found that the loss of *stx1* was more common in non-O157 *E. coli* isolates (45%) compared to O157 (15%) upon sub-cultivation. Unstable Shiga toxin detection has been demonstrated in Enterohaemorrhagic *E. coli* (EHEC) and atypical Enterohaemorrhagic *E. coli* (aEHEC) O26:H11 with 10% to 14% of isolates losing *stx2* and aEHEC conversion to EHEC upon first sub-cultivation [69]. Some studies have also shown that *stx* genes can also be acquired, as *stx2* encoding prophages were found to integrate into aEHEC O26:H11 as hot spots within the genome [69]. Others have attributed the acquisition of virulence in STEC to the integration of prophages carrying virulence determinants [70], which play a major role in the gain and loss of genes through lysogenic or decay processes [71]. Genes associated with Stx-conversion are mostly encoded by the lambdoid phages [72].

There is also the possibility that STEC cultures consisted of a mixture of isolates that possessed or lacked *stx* genes. Although the likelihood of this would be reduced by repetitive sub-culturing, this possibility can not be completely eliminated. Thus, a random selection of colonies that exhibit this variability could account for the inconsistent confirmation of the presence of stx between PCR detection and WGS annotation. The inconsistent *stx* results in this study perhaps reflect the unstable nature of Stx-encoding phages upon sub-culturing or other mechanisms that are yet to be understood and require further investigation.

A low proportion of *eaeA* (15%) genes in non-O157 *E. coli* agree with previous findings, where a lower percentage of *eaeA* was observed in non-O157 strains [58]. However, Stanford et al. [61] and Cernicchiaro et al. [73] found higher percentages of *eaeA* in non-O157 isolates from Western Canada and the United States, respectively. *EaeA* encodes intimin, which promotes bacterial attachment to host intestinal mucosa cells [74] and enhances infection by colonizing the intestinal wall. The *eaeA* gene was detected in O26:H11 (n = 2) and O156:25 indicating they harbor the pathogenicity island, the Locus for Enterocyte Effacement (LEE). However, the presence of *eaeA* may only suggest an additional virulence factor for these strains as LEE-negative STEC strains can produce adhesins other than *eaeA* [75]. Additional adhesins genes (*tox B*; 8.3%, *ecpA*; 91.7% and *csgA*; 100%), which are encoded outside the LEE region and identified in this study, could represent important adherence factors in the LEE-negative STEC strains. Hemolysin is thought to play an important role in *E. coli* infections in synergy with other virulence genes [76], causing red blood cell lysis and activating apoptosis [77]. All non-O157 isolates that tested positive for either or both *stx1* and *stx2* genes in this study possessed the *hlyA* gene. The results of this study highlight the need for food safety controls to be established to mitigate the dissemination of these potential virulent strains from farm to food products and surface/ground water, which may serve as vehicles of transmission to consumers.

### 4.2. Antimicrobial Susceptibility

Compared to other antimicrobials, a higher proportion of tetracycline resistance in this study is in agreement with other South African studies [24,26] for STEC and non STEC O157:H7 isolates from livestock, respectively. This may be a reflection of the abundant use of tetracycline as a growth promoter in cattle feed and as a disease control agent. Furthermore, tetracycline resistance is transferred by mobile genetic elements that are widely distributed across different bacterial genera [78]. Although *tetA* was present in higher proportions compared to *tetB*, a strong association with phenotypic resistance to tetracycline was observed for *tetB*. All isolates (11.3%) having the *tetB* gene expressed phenotypic resistance. Conversely, 16 of 25 (64%) *tetA*-possessing isolates did not express phenotypic resistance. This observation agrees with previous studies where the majority of *E. coli* isolates that exhibited tetracycline resistance encoded *tetB* [79,80]. Of eleven MDR isolates, four isolates harboured *blaTEM-1* and demonstrated resistance to ampicillin, indicating a strong genotype:phenotype association. Shaikh et al. [81] highlighted growing concerns regarding the current rise in extended-spectrum beta-lactamase-resistant Enterobacteriaceae. Chloramphenicol usage in food-producing animals has been banned since the 1990s in South Africa [82]. However, phenotypic resistance was observed in three isolates. No gene to chloramphenicol-resistance was detected in these isolates. Similarly, phenotypic resistance for streptomycin was not related to the presence of (*aadA1*), suggesting that resistance might have been conferred by other resistance genes (*aadA2*, *aadA5* and *aadA23*) not tested in this study or as a result of genes coding for unknown resistance mechanisms. In addition, there are other forms of resistance mechanisms such as intrinsic resistance driven by efflux pumps, adaptive resistance and regulatory mutations [13,83]. The presence of these resistance genes in non-O157 *E. coli* isolates from cattle faecal samples may represent a reservoir of resistance genes that are transferable to other bacteria in the gut and subsequent dissemination into the environment. Antimicrobial resistance patterns across the three different regions of this study indicate that MDR non-O157 *E coli* isolates were predominately from the Rooigrond region. This region has both beef and dairy production systems, raising the possibility of contamination of both meat and milk, making it important to gain a better understanding of AMR profile in non-O157 *E coli* in this region is required.

### 4.3. Biofilm Formation and Its Associated Genes 

To the best of our knowledge, no biofilm data on antimicrobial-resistant non-O157 STEC strains have been previously described. Most non-O157 *E. coli* isolates in this study were able to adhere and form biofilms on polystyrene. Biofilm-forming ability of these isolates varied, suggesting a high diversity and strain specificity [17,84]. Biofilm formation was more evident at 48 h compared to 24 h and 72 h at 22 °C and 37 °C, respectively. This observation corroborates the findings of Biscola et al. [85], where strong biofilm of non-O157 at 28 °C for 48 h was observed. Similarly, strong and intermediate biofilm detection at 22 °C in this study compared to 37 °C agree with the findings of Wang et al. [17]. All non-O157 *E. coli* isolates in this study possessed the genes (*crl* and *csgA* and *csgD*) associated with curli expression during biofilm formation. However, the biofilm-forming ability could not be directly linked to the presence of these genes as 29.7% and 62.5% of the isolates that possessed these genes *crl* and *csgA* and *csgD* formed weak biofilms at 22 °C and 37 °C, respectively. Perhaps these biofilm-forming genes were either not functional in these isolates or they do not fully account for the mechanisms of biofilm formation [86]. Biscola et al. [85] also found that not all curli-positive strains developed the biofilm-forming phenotype in vitro. 

RNA polymerase sigma factor S (*rpoS*) gene, encoding a stress response sigma factor is required for transcription in bacteria and regulates the response of cells to general environmental stress. This gene was present in 82.8% of non-O157 *E. coli* in this study and was a strong predictor of biofilm-forming ability of isolates at 22 °C and 37 °C. According to White-Ziegler et al. [87], *rpoS* is a thermoregulator that is expressed more at 23 °C than at 37 °C and impacts biofilm-forming genes including *csgA* and *csgD.* Both of these genes were detected in this study and these isolates exhibited robust biofilm formation at 22 °C than at 37 °C, indicating that temperature was an important factor to which *rpoS*-regulated genes are activated in different environmental conditions. Finally, the type 1 fimbriae gene (*fimH*), together with autotransporter genes (*ehaA^α^*, *ehaA^β^* and *flu*) detected in this study were not statistically linked to biofilm formation. Therefore, further exploration of the functions of these genes in biofilm formation is needed. MDR isolates with a strong ability to adhere to polystyrene and form biofilm is a cause for concern as most of the cells in biofilm communities are in a state of metabolic stasis [88] and exhibit enhanced resistance to antimicrobials [89]. Furthermore, the surrounding exopolysaccharide matrix also acts as a barrier, preventing antimicrobials from contacting targeted bacteria [90].

### 4.4. PFGE

The fact that PFGE identified 37 unique patterns from 59 screened isolates reflects the diversity of non-O157 *E*. *coli.* Five isolates, which were not digested by *XbaI* despite efforts to optimize the procedure, were deemed untypable, as observed in other STEC studies [91]. A high diversity of non-O157 *E. coli* in cattle faeces using *XbaI* PFGE typing has previously been reported in France [92]. This high diversity indicates that cattle are an important animal reservoir for the emergence of potentially pathogenic non-O157 *E. coli*.

### 4.5. Whole Genome Sequence Analysis

The lipopolysaccharide (O) antigen is a highly variable region of the outer membrane of Gram-negative bacteria [93]. Together with the flagellar (H) antigen, the O-antigen is used in the identification of pathogenic *E. coli* strains [94]. An O-antigen locus that has a unique arrangement of genes compared with a novel reference allele in the EcOH database is termed a novel O-locus [37]. In silico, WGS identified diverse non-O157 *E coli* serotypes harbouring a broad range of VFs, antimicrobial resistant determinants and different plasmids types. Serotypes such as O156:H25 (n = 1), O17:H18 (n = 1), O163:H19 (n = 1), O116:H11 (n = 1), wzx-Onovel5:H19 (n = 2) and O26:H11 (n = 3) possessed more VFs than other serotypes predicted. Amongst these, O26:H11 is the most commonly reported non-O157 *E. coli* in human infections [3]. These factors range from cell-associated adhesins that can mediate binding to different surfaces to secreted toxins. Interestingly, WGS analysis indicated that the two novel-O (wzx-Onovel5) isolates possessing *stx2* were clones contrary to PFGE-based results. Serotype O156:H25, an MDR strain characterized in this study via phenotypic/genotypic-based method and WGS, has been associated with *E. coli* strains that colonize cattle in Germany [95]. According to the Comprehensive Antibiotic Resistance Database [38], the antimicrobial resistance gene *udg* detected at 79.2% of isolates, is responsible for the synthesis of lipid A, which offers resistance to cationic antimicrobial peptides and is a common gene in many *E. coli* isolates.

Plasmids are thought to play a vital role in the mobility of antimicrobial resistance determinants via horizontal gene transfer between different bacterial species [96]. The *ColRNAI* replicon was detected in a large proportion of the isolates (50.0%), a level similar to that reported by others [97]. Plasmids of the Inc-family are frequently associated with MDR and virulence determinants in *E. coli* [98]. Thirteen Inc-related replicons were detected in this study especially the *IncFIB* (AP001918; 62.5%) compared to other Inc plasmids. Interestingly, O156:H25 an MDR isolate carried only one plasmid type (IncY), inferring resistance was chromosome-mediated as detected via PCR and WGS. 

These potential pathogenic strains predicted by means of WGS may have remained uncharacterized using a traditional method such as serology- and PCR-based (virulence, antimicrobial and plasmid) typing methods. Thus, in silico WGS serotyping offers a rapid and accurate approach to identifying potential pathogenic strains. Use of WGS also has an advantage over serology-based typing methods, which frequently fail type isolates [37]. In addition, WGS offers a higher resolution for *stx*-subtyping [99] compared to PCR and a higher level of strain discrimination [100] than PFGE. Considering that WGS offers a more comprehensive and detailed description of bacterial strains it can be allied with traditional typing methods of non-O157 *E. coli*.

## 5. Conclusions

These findings indicate that cattle from the North West of South Africa harboured genetically diverse, virulent, antimicrobial-resistant and novel pathotypes of non-O157 STEC that formed biofilms. Biofilm-forming ability may increase the persistence and dissemination of these pathogens in the environment and possibly subsequent risk of contamination or infection. Furthermore, biofilm-forming AMR isolates could persist in the environment and lead to the spread of AMR genes to pathogenic or non-pathogenic bacteria that can in turn transfer resistance to human or animal pathogens. Finally, WGS is a powerful tool to discriminate and predict the pathogenicity of a wide range of *E. coli* strains from cattle faeces and has an important role in assessing food safety and human health risks of non-O157 STEC.

## Figures and Tables

**Table 1:** Number of isolates for Shiga-toxins and other virulence factors genes detected by PCR.

Sampling Region	Gene					
	*hlyA*	*eaeA*	*stx1*	*stx2*	*stx1/stx2*	*stx2a*
Koster dairy n = 20	11	4	7	12	3	0
Vryburg beef n = 10	3	0	1	6	1	0
Rooigrond dairy n = 22	14	4	1	18	1	2
Rooigrond beef n = 28	24	4	11	24	11	9
% isolates positive for each gene/both	65	15	25	75	20	13.8

n = number of isolates.

**Table 2:** Phenotypic resistance and antimicrobial resistance genes associated with non-O157 *E. coli* isolates.

Sampling Region	Number of Isolates with Antimicrobial Resistance/Resistance Genes							
	AMS	AMP/*blaTEM-1*	STR/*aadA1*	CHL/*catA1*	TET/*tetA*/*tetB*	NAL	NOR	SXT
Koster dairy n = 20	0	0	0	0/0	2/2/0	0	0	0
Vryburg beef n = 10	0	0	0	0/0	1/3/0	0	0	0
Rooigrond dairy n = 22	1	2/2	6/0	2/0	8/11/5	0	0	2
Rooigrond beef n = 28	0	2/2	5/2	1/0	5/9/4	1	1	2
% isolate positive for phenotypic/genotypic resistance	1.3	5/5	13.7/2.5	3.7/0	20/31.3/11.3	1.3	1.3	5

AMS: Ampicillin-sulbactam, AMP: Ampicillin, STR: Streptomycin, CHL: Chloramphenicol, TET: Tetracycline, NAL: Nalidixic acid, NOR: Norfloxacin and SXT: Trimethoprim-sulfamethoxazole. *blaTEM-1*: beta-lactams resistance gene, *tetA*/*B*: tetracycline resistance gene, *aadA1*: streptomycin resistance gene, *catA1*: chloramphenicol resistance gene, n= number of isolates, phenotypic resistance: disc diffusion and genotypic resistance: resistance gene presence.

**Table 3:** Multidrug resistance isolates.

Sampling Region	Source	Number of Isolates	Resistance Phenotype	Intermediate Phenotype
Rooigrond	Dairy	1	STR-TET-AMP-CHL-SXT	AMS-AMC
		1	STR-TET-AMP-CHL-AMS	AMC
		1	STR-TET-SXT	AMC
		4	STR-TET	-
	Beef	2	STR-TET	AMC
		1	STR-TET-AMP-SXT	-
		1	STR-TET-AMP-CHL-SXT-NAL-NOR	-

STR: Streptomycin, TET: Tetracycline, AMP: Ampicillin, CHL: Chloramphenicol, AMS: Ampicillin-sulbactam, SXT: Trimethoprim-sulfamethoxazole, NAL: Nalidixic acid, NOR: Norfloxacin and AMC: Amoxicillin-clavulanate.

**Table 4:** Biofilm-forming ability of non-O157 *E. coli* isolates on polystyrene and number of isolates positive for biofilm forming genes in isolates that were positive for Shiga toxin genes and multi-drug resistant non-STEC.

Sampling Region	Biofilm-Forming Ability				Biofilm-Forming Genes							
	Strong Biofilm (OD 0.32)		Weak Biofilm (OD 0.082)		*csgA*	*csgD*	*crl*	*fimH*	*flu*	*rpoS*	*ehaA^α^*	*ehaA^β^*
	22 °C	37 °C	22 °C	37 °C								
Koster dairy n = 16	10	5	6	11	16	16	16	15	9	13	11	9
Vryburg beef n = 6	5	5	1	1	6	6	6	6	4	6	4	1
Rooigrond dairy n = 18	7	3	11	15	18	18	18	16	14	10	9	5
Rooigrond beef n = 24	23	11	1	13	24	24	24	24	22	24	15	5
% Isolates indicating biofilm-forming ability and each biofilm-forming gene	70.3	37.5	29.7	62.5	100	100	100	95.3	76.5	82.8	60.9	31.2

n = number of isolates; *csgA*: Curli structural subunit, *csgA*: Curli regulator D, *crl*: Curli regulator, *fimH*: Type 1 fimbriae, *flu*: Antigen 43 autotrasporter protein, *rpoS*: DNA-binding proteins for regulating *csgD*, *ehaA^α^*: Eha passenger and *ehaA^β^*: Eha translocation domain.

**Table 5:** Sequence annotation results for non-O157 *E. coli* serotype determinants (O- and H-antigen sequences) and *stx* genes.

Sampling Region	Isolate Number	O-Type	H-Type	*stx* Genes Based on PCR Detection	*stx* Genes Based on Annotated Results
Koster dairy	3	O99	H9	none	none
	11	O156	H25	1	1
	12	O108	H2	1&2	none
	14	O136	H30	1	none
	15	O99	H9	2	none
Vryburg beef	22	wzx-Onovel24	H20	none	none
	25	O140	H21	1&2	none
	30	O102	H4	2	none
Rooigrond dairy	32	O129	H23	2	none
	37	O17	H18	2	2
	38	O76	H34	2	none
	42	O26	H11	1&2	none
	50	O129	H23	2	none
	69	O26	H11	2	none
	72	O26	H11	2	none
	76	O163	H19	2	none
	77	O40	H19	2	2
	80	O22	H21	none	none
Rooigrond beef	56	O154	H10	1&2	none
	60	O116	H21	2	2
	64	*wzx*-Onovel5	H19	1&2	2
	65	*wzx*-Onovel5	H19	2	2
	67	O87	H7	1&2	none
	68	O129	H21	1&2	2

None: no *stx* gene detected.

## Supplementary Materials

The following are available online at https://www.mdpi.com/2076-2607/7/8/272/s1, Figure S1: A flow chart illustrating bacterial characterization, Figure S2: Multiplex gel image of amplicons indicating no detection of *stx2* (gel B). M indicates molecular marker 1kb plus, C+ the positive control, C− the negative control and isolate number (40–79), Table S1: PCR primer sequences, PCR product sizes and references, Table S2: Reference values used to classify isolates as susceptible, intermediate resistant and resistant, Table S3: Non-O157 *E. coli* isolates sequenced in this study Table S4: Annotation of virulence factors for non-O157 *E. coli* isolates, Table S5: Annotation results of antimicrobial resistance for non-O157 *E. coli*, Table S6: Annotated results of plasmids search for non-O157 *E. coli*.

## References

[1] Marejková M., Bláhová K., Janda J., Fruth A., Petráš P. (2013). Enterohemorrhagic *Escherichia coli* as causes of hemolytic uremic syndrome in the Czech Republic. PLoS ONE.

[2] Brooks J., Sowers E., Wells J., Greene K., Griffin P., Hoekstra R., Strockbine N. (2005). Non-O157 Shiga toxin–producing *Escherichia coli* infections in the United States, 1983–2002. J. Infect. Dis..

[3] Luna-Gierke R., Griffin P., Gould L., Herman K., Bopp C., Strockbine N., Mody R. (2014). Outbreaks of non-O157 Shiga toxin-producing *Escherichia coli* infection: USA. Epidemiol. Infect..

[4] Tarr P.I., Gordon C.A., Chandler W.L. (2005). Shiga-toxin-producing *Escherichia coli* and haemolytic uraemic syndrome. Lancet.

[5] Karmali M.A. (2016). Emerging public health challenges of Shiga toxin–producing *Escherichia coli* related to changes in the pathogen, the population, and the environment. Clin. Infect. Dis..

[6] Hussein H. (2007). Prevalence and pathogenicity of Shiga toxin-producing *Escherichia coli* in beef cattle and their products. J. Anim. Sci..

[7] Kosek M., Bern C., Guerrant R.L. (2003). The global burden of diarrhoeal disease, as estimated from studies published between 1992 and 2000. Bull. World Health Organ..

[8] Wong C.S., Brandt J.R. (2002). Risk of hemolytic uremic syndrome from antibiotic treatment of *Escherichia coli* O157:H7 colitis. JAMA.

[9] Scheutz F., Teel L., Beutin L., Piérard D., Buvens G., Karch H., Mellmann A., Caprioli A., Tozzoli R., Morabito S. (2012). Multicenter evaluation of a sequence-based protocol for subtyping Shiga toxins and standardizing Stx nomenclature. J. Clin. Microbiol..

[10] Johannes L., Römer W. (2010). Shiga toxins-from cell biology to biomedical applications. Nat. Rev. Microbiol..

[11] Donlan R.M. (2002). Biofilms: Microbial life on surfaces. Emerg. Infect. Dis..

[12] Neelakantan P., Romero M., Vera J., Daood U., Khan A., Yan A., Cheung G. (2017). Biofilms in Endodontics—Current status and future directions. Int. J. Mol. Sci..

[13] Hughes G., Webber M.A. (2017). Novel approaches to the treatment of bacterial biofilm infections. Br. J. Pharmacol..

[14] Paton J.C., Paton A.W. (1998). Pathogenesis and diagnosis of Shiga toxin-producing *Escherichia coli* infections. Clin. Microbiol. Rev..

[15] Chua S.L., Liu Y., Yam J.K.H., Chen Y., Vejborg R.M., Tan B.G.C., Kjelleberg S., Tolker-Nielsen T., Givskov M., Yang L. (2014). Dispersed cells represent a distinct stage in the transition from bacterial biofilm to planktonic lifestyles. Nat. Commun..

[16] Uhlich G.A., Chen C.Y., Cottrell B.J., Nguyen L.H. (2014). Growth media and temperature effects on biofilm formation by serotype O157:H7 and non-O157 Shiga toxin-producing *Escherichia coli*. FEMS Microbiol. Lett..

[17] Wang J., Stanford K., McAllister T.A., Johnson R.P., Chen J., Hou H., Zhang G., Niu Y.D. (2016). Biofilm Formation, Virulence Gene Profiles, and Antimicrobial Resistance of Nine Serogroups of Non-O157 Shiga Toxin-Producing *Escherichia coli*. Foodborne Pathog. Dis..

[18] Ma Z., Bumunang E.W., Stanford K., Bie X., Niu Y.D., McAllister T.A. (2019). Biofilm Formation by Shiga Toxin-Producing *Escherichia coli* on Stainless Steel Coupons as Affected by Temperature and Incubation Time. Microorganisms.

[19] Hall M.R., McGillicuddy E., Kaplan L.J. (2014). Biofilm: Basic principles, pathophysiology, and implications for clinicians. Surg. Infect..

[20] Wilson M. (2001). Bacterial biofilms and human disease. Sci. Prog..

[21] Frank J.F. (2001). Microbial attachment to food and food contact surfaces. Adv. Food Nutr. Res..

[22] Ntuli V., Njage P.M.K., Buys E.M. (2016). Characterization of *Escherichia coli* and other Enterobacteriaceae in producer-distributor bulk milk. J. Dairy Sci..

[23] Caine L.-A., Nwodo U.U., Okoh A.I., Ndip R.N., Green E. (2014). Occurrence of virulence genes associated with diarrheagenic *Escherichia coli* isolated from raw cow’s milk from two commercial dairy farms in the Eastern Cape Province, South Africa. Int. J. Environ. Res. Public Health.

[24] Iweriebor B.C., Iwu C.J., Obi L.C., Nwodo U.U., Okoh A.I. (2015). Multiple antibiotic resistances among Shiga toxin producing *Escherichia coli* O157 in feces of dairy cattle farms in Eastern Cape of South Africa. BMC Microbiol..

[25] Phokela P.T., Ateba C.N., Kawadza D.T. (2011). Assessing antibiotic resistance profiles in *Escherichia coli* and Salmonella species from groundwater in the Mafikeng area, South Africa. Afr. J. Microbiol. Res..

[26] Ateba C.N., Bezuidenhout C.C. (2008). Characterisation of *Escherichia coli* O157 strains from humans, cattle and pigs in the North-West Province, South Africa. Int. J. Food Microbiol..

[27] Anbazhagan D., Mui W.S., Mansor M., Yan G.O.S., Yusof M.Y., Sekaran S.D. (2011). Development of conventional and real-time multiplex PCR assays for the detection of nosocomial pathogens. Braz. J. Microbiol..

[28] Morin N.J., Gong Z., Li X.-F. (2004). Reverse transcription-multiplex PCR assay for simultaneous detection of *Escherichia coli* O157:H7, *Vibrio cholerae* O1, and *Salmonella Typhi*. Clin.Chem..

[29] Momtaz H., Rahimi E., Moshkelani S. (2012). Molecular detection of antimicrobial resistance genes in *E. coli* isolated from slaughtered commercial chickens in Iran. Vet. Med..

[30] Paton A., Paton J. (1998). Detection and Characterization of Shiga Toxigenic *Escherichia coli* by Using Multiplex PCR Assays for *stx1*, *stx2*, *eaeA*, Enterohemorrhagic *E. coli hlyA*, *rfb* O111, and *rfb* O157. J. Clin. Microbiol..

[31] CLSI (2017). Performance Standards for Antimicrobial Susceptibility Testing.

[32] Stepanović S., Vuković D., Dakić I., Savić B., Švabić-Vlahović M. (2000). A modified microtiter-plate test for quantification of staphylococcal biofilm formation. J. Microbiol. Methods.

[33] Ribot E.M., Fair M., Gautom R., Cameron D., Hunter S., Swaminathan B., Barrett T.J. (2006). Standardization of pulsed-field gel electrophoresis protocols for the subtyping of *Escherichia coli* O157:H7, *Salmonella*, and *Shigella* for PulseNet. Foodborne Pathog. Dis..

[34] CDC (2013). Standard operating procedure for PulseNet PFGE of Escherichia coli O157:H7, Escherichia coli non-O157 (STEC), Salmonella serotypes, Shigella sonnei and Shigella flexneri.

[35] Bankevich A., Nurk S., Antipov D., Gurevich A.A., Dvorkin M., Kulikov A.S., Lesin V.M., Nikolenko S.I., Pham S., Prjibelski A.D. (2012). SPAdes: A new genome assembly algorithm and its applications to single-cell sequencing. J. Comput. Biol..

[36] Seemann T. (2014). Prokka: Rapid prokaryotic genome annotation. Bioinformatics.

[37] Ingle D.J., Valcanis M., Kuzevski A., Tauschek M., Inouye M., Stinear T., Levine M.M., Robins-Browne R.M., Holt K.E. (2016). In silico serotyping of *E. coli* from short read data identifies limited novel O-loci but extensive diversity of O:H serotype combinations within and between pathogenic lineages. Microb. Genom..

[38] Jia B., Raphenya A.R., Alcock B., Waglechner N., Guo P., Tsang K.K., Lago B.A., Dave B.M., Pereira S., Sharma A.N. (2016). CARD 2017: expansion and model-centric curation of the comprehensive antibiotic resistance database. Nucleic Acids Res..

[39] Easton D.M., Totsika M., Allsopp L.P., Phan M.-D., Idris A., Wurpel D.J., Sherlock O., Zhang B., Venturini C., Beatson S.A. (2011). Characterization of EhaJ, a new autotransporter protein from enterohemorrhagic and enteropathogenic *Escherichia coli*. Front. Microbiol..

[40] Leyton D.L., Sloan J., Hill R.E., Doughty S., Hartland E.L. (2003). Transfer region of pO113 from enterohemorrhagic *Escherichia coli*: Similarity with R64 and identification of a novel plasmid-encoded autotransporter, EpeA. Infect. Immun..

[41] Van der Woude M.W., Henderson I.R. (2008). Regulation and function of Ag43 (flu). Annu. Rev. Microbiol..

[42] Batisson I., Guimond M.-P., Girard F., An H., Zhu C., Oswald E., Fairbrother J.M., Jacques M., Harel J. (2003). Characterization of the novel factor paa involved in the early steps of the adhesion mechanism of attaching and effacing *Escherichia coli*. Infect. Immun..

[43] Rendón M.A.A., Saldaña Z., Erdem A.L., Monteiro-Neto V., Vázquez A., Kaper J.B., Puente J.L., Girón J.A. (2007). Commensal and pathogenic *Escherichia coli* use a common pilus adherence factor for epithelial cell colonization. Proc. Natl. Acad. Sci. USA.

[44] Tatsuno I., Horie M., Abe H., Miki T., Makino K., Shinagawa H., Taguchi H., Kamiya S., Hayashi T., Sasakawa C. (2001). *toxB* gene on pO157 of enterohemorrhagic *Escherichia coli* O157:H7 is required for full epithelial cell adherence phenotype. Infect. Immun..

[45] Hammar M.R., Arnqvist A., Bian Z., Olsén A., Normark S. (1995). Expression of two csg operons is required for production of fibronectin and congo red binding curli polymers in *Escherichia coli* K 12. Mol. Microbiol..

[46] Kim K.S. (2002). Strategy of *Escherichia coli* for crossing the blood-brain barrier. J. Infect. Dis..

[47] Schembri M.A., Christiansen G., Klemm P. (2001). FimH mediated autoaggregation of *Escherichia coli*. Mol. Microbiol..

[48] Torres A.G., Payne S.M. (1997). Haem iron transport system in enterohaemorrhagic *Escherichia coli* O157:H7. Mol. Microbiol..

[49] Dutta P.R., Cappello R., Navarro-García F., Nataro J.P. (2002). Functional comparison of serine protease autotransporters of Enterobacteriaceae. Infect. Immun..

[50] Cortajarena A.L., Goñi F.M., Ostolaza H. (2003). A receptor-binding region in *Escherichia coli* α-haemolysin. J. Biol. Chem..

[51] Nešić D., Hsu Y., Stebbins C.E. (2004). Assembly and function of a bacterial genotoxin. Nature.

[52] Ménard L.-P., Dubreuil J.D. (2002). Enteroaggregative *Escherichia coli* heat-stable enterotoxin 1 (EAST1): A new toxin with an old twist. Crit. Rev. Microbiol..

[53] Cergole-Novella M.C., Nishimura L.S., Dos Santos L.F., Irino K., Vaz T.M.I., Bergamini A.M., Guth B.E.C. (2007). Distribution of virulence profiles related to new toxins and putative adhesins in Shiga toxin-producing *Escherichia coli* isolated from diverse sources in Brazil. FEMS Microbiol. Lett..

[54] Daniell S.J., Kocsis E., Morris E., Knutton S., Booy F.P., Frankel G. (2003). 3 D structure of EspA filaments from enteropathogenic *Escherichia coli*. Mol. Microbiol..

[55] Fairbrother J., Nadeau E. (2006). *Escherichia coli*: On-farm contamination of animals. Rev. Sci. Tech..

[56] Allende A., Monaghan J. (2015). Irrigation water quality for leafy crops: A perspective of risks and potential solutions. Int. J. Environ. Res. Public Health.

[57] Mainga A.O., Cenci-Goga B.T., Malahlela M.N., Tshuma T., Kalake A., Karama M. (2018). Occurrence and characterization of seven major Shiga toxin-producing *Escherichia coli* serotypes from healthy cattle on cow–calf operations in South Africa. Zoonoses Public Health.

[58] Tahamtan Y., Hayati M., Namavari M. (2010). Prevalence and distribution of the *stx1*, *stx2* genes in Shiga toxin producing *E. coli* (STEC) isolates from cattle. Iran. J. Microbiol..

[59] Keen J.E., Wittum T.E., Dunn J.R., Bono J.L., Durso L.M. (2006). Shiga-toxigenic *Escherichia coli* O157 in agricultural fair livestock, United States. Emerg. Infect. Dis..

[60] Stanford K., Johnson R.P., Alexander T.W., McAllister T.A., Reuter T. (2016). Influence of season and feedlot location on prevalence and virulence factors of seven serogroups of *Escherichia coli* in feces of western-Canadian slaughter cattle. PLoS ONE.

[61] Anjum M., Jones E., Morrison V., Tozzoli R., Morabito S., Toth I., Nagy B., Smith G., Aspan A., Nielsen E. (2014). Use of virulence determinants and seropathotypes to distinguish high-and low-risk *Escherichia coli* O157 and non-O157 isolates from Europe. Epidemiol. Infect..

[62] Martinez-Castillo A., Quirós P., Navarro F., Miró E., Muniesa M. (2013). Shiga toxin 2-encoding bacteriophages in human fecal samples from healthy individuals. Appl. Environ. Microbiol..

[63] Soborg B., Lassen S., Muller L., Jensen T., Ethelberg S., Mølbak K., Scheutz F. (2013). A verocytotoxin-producing *E. coli* outbreak with a surprisingly high risk of haemolytic uraemic syndrome, Denmark, September-October 2012. Euro Surveill..

[64] Persson S., Olsen K.E., Ethelberg S., Scheutz F. (2007). Subtyping method for *Escherichia coli* Shiga toxin (verocytotoxin) 2 variants and correlations to clinical manifestations. J. Clin. Microbiol..

[65] Fuller C.A., Pellino C.A., Flagler M.J., Strasser J.E., Weiss A.A. (2011). Shiga toxin subtypes display dramatic differences in potency. Infect. Immun..

[66] Gyles C. (2007). Shiga toxin-producing: An overview. J. Nnimal. Sci..

[67] Karch H., Meyer T., Rüssmann H., Heesemann J. (1992). Frequent loss of Shiga-like toxin genes in clinical isolates of *Escherichia coli* upon subcultivation. Infect. Immun..

[68] Joris M.-A., Verstraete K., De Reu K., De Zutter L. (2011). Loss of *vtx* genes after the first subcultivation step of verocytotoxigenic *Escherichia coli* O157 and non-O157 during isolation from naturally contaminated fecal samples. Toxins.

[69] Bielaszewska M., Prager R., Köck R., Mellmann A., Zhang W., Tschäpe H., Tarr P.I., Karch H. (2007). Shiga toxin gene loss and transfer in vitro and in vivo during enterohemorrhagic *Escherichia coli* O26 infection in humans. Appl. Environ. Microbiol..

[70] Busby B., Kristensen D.M., Koonin E.V. (2013). Contribution of phage-derived genomic islands to the virulence of facultative bacterial pathogens. Environ. Microbiol..

[71] Brüssow H., Canchaya C., Hardt W.-D. (2004). Phages and the evolution of bacterial pathogens: from genomic rearrangements to lysogenic conversion. Microbiol. Mol. Biol. Rev..

[72] Schmidt H. (2001). Shiga-toxin-converting bacteriophages. Res. Microbiol..

[73] Cernicchiaro N., Cull C.A., Paddock Z.D., Shi X., Bai J., Nagaraja T.G., Renter D.G. (2013). Prevalence of Shiga toxin–producing *Escherichia coli* and associated virulence genes in feces of commercial feedlot cattle. Foodborne Pathog. Dis..

[74] Vallance B., Finlay B. (2000). Exploitation of host cells by enteropathogenic *Escherichia coli*. Proc. Natl. Acad. Sci. USA.

[75] Bettelheim K.A. (2007). The non-O157 Shiga-toxigenic (verocytotoxigenic) *Escherichia coli*; under-rated pathogens. Crit. Rev. Microbiol..

[76] Boerlin P., McEwen S.A., Boerlin-Petzold F., Wilson J.B., Johnson R.P., Gyles C.L. (1999). Associations between virulence factors of Shiga toxin-producing *Escherichia coli* and disease in humans. J. Clin. Microbiol..

[77] Bien J., Sokolova O., Bozko P. (2012). Role of uropathogenic *Escherichia coli* virulence factors in development of urinary tract infection and kidney damage. Int. J. Nephrol..

[78] Sunde M., Norström M. (2006). The prevalence of, associations between and conjugal transfer of antibiotic resistance genes in *Escherichia coli* isolated from Norwegian meat and meat products. J. Antimicrob. Chemother..

[79] Gow S.P., Waldner C.L., Harel J., Boerlin P. (2008). Associations between antimicrobial resistance genes in fecal generic *Escherichia coli* isolates from cow-calf herds in western Canada. Appl. Environ. Microbiol..

[80] Sawant A.A., Hegde N.V., Straley B.A., Donaldson S.C., Love B.C., Knabel S.J., Jayarao B.M. (2007). Antimicrobial-resistant enteric bacteria from dairy cattle. Appl. Environ. Microbiol..

[81] Shaikh S., Fatima J., Shakil S., Rizvi S.M.D., Kamal M.A. (2015). Antibiotic resistance and extended spectrum beta-lactamases: Types, epidemiology and treatment. Saudi J. Biol. Sci..

[82] Henton M.M., Eagar H.A., Swan G.E., Van Vuuren M. (2011). Part VI. Antibiotic management and resistance in livestock production. S. Afr. Med. J..

[83] Sandoval-Motta S., Aldana M. (2016). Adaptive resistance to antibiotics in bacteria: a systems biology perspective. Wiley Interdiscip. Rev. Syst. Biol. Med..

[84] Naves P., Del Prado G., Huelves L., Gracia M., Ruiz V., Blanco J., Rodríguez-Cerrato V., Ponte M., Soriano F. (2008). Measurement of biofilm formation by clinical isolates of *Escherichia coli* is method-dependent. J. Appl. Microbiol..

[85] Biscola F.T., Abe C.M., Guth B.E.C. (2011). Determination of adhesin gene sequences in, and biofilm formation by, O157 and non-O157 Shiga toxin-producing *Escherichia coli* strains isolated from different sources. Appl. Environ. Microbiol..

[86] López D., Vlamakis H., Kolter R. (2010). Biofilms. Cold Spring Harb. Perspect. Biol..

[87] White-Ziegler C.A., Um S., Perez N.M., Berns A.L., Malhowski A.J., Young S. (2008). Low temperature (23 C) increases expression of biofilm-, cold-shock-and RpoS-dependent genes in *Escherichia coli* K-12. Microbiology.

[88] Spoering A.L., Lewis K. (2001). Biofilms and planktonic cells of *Pseudomonas aeruginosa* have similar resistance to killing by antimicrobials. J. Bacteriol..

[89] Jouenne T., Mor A., Bonato H., Junter G. (1998). Antibacterial activity of synthetic dermaseptins against growing and non-growing *Escherichia coli* cultures. J. Antimicrob. Chemother..

[90] Jolivet-Gougeon A., Bonnaure-Mallet M. (2014). Biofilms as a mechanism of bacterial resistance. Drug Discov. Today Technol..

[91] Apun K., Chang P., Sim E., Micky V. (2006). Clonal diversity of *Escherichia coli* isolates from marketed beef in East Malaysia. World J. Microbiol. Biotechnol..

[92] Bibbal D., Loukiadis E., Kérourédan M., Ferré F., Dilasser F., de Garam C.P., Cartier P., Oswald E., Gay E., Auvray F. (2015). Prevalence of carriage of Shiga toxin-producing *Escherichia coli* serotypes O157:H7, O26:H11, O103:H2, O111:H8, and O145:H28 among slaughtered adult cattle in France. Appl. Environ. Microbiol..

[93] Iguchi A., Iyoda S., Kikuchi T., Ogura Y., Katsura K., Ohnishi M., Hayashi T., Thomson N.R. (2014). A complete view of the genetic diversity of the *Escherichia coli* O-antigen biosynthesis gene cluster. DNA Res..

[94] DebRoy C., Fratamico P.M., Yan X., Baranzoni G., Liu Y., Needleman D.S., Tebbs R., O’Connell C.D., Allred A., Swimley M. (2016). Comparison of O-antigen gene clusters of all O-serogroups of *Escherichia coli* and proposal for adopting a new nomenclature for O-typing. PLoS ONE.

[95] Geue L., Menge C., Eichhorn I., Semmler T., Wieler L.H., Pickard D., Berens C., Barth S.A. (2017). Evidence for Contemporary Switching of the O-Antigen Gene Cluster between Shiga Toxin-Producing *Escherichia coli* Strains Colonizing Cattle. Front. Microbiol..

[96] Carattoli A. (2013). Plasmids and the spread of resistance. Int. J. Med. Microbiol..

[97] Ahmed S., Olsen J.E., Herrero-Fresno A. (2017). The genetic diversity of commensal *Escherichia coli* strains isolated from non-antimicrobial treated pigs varies according to age group. PLoS ONE.

[98] Johnson T.J., Nolan L.K. (2009). Pathogenomics of the virulence plasmids of *Escherichia coli*. Microbiol. Mol. Biol. Rev..

[99] Holmes A., Allison L., Ward M., Dallman T.J., Clark R., Fawkes A., Murphy L., Hanson M. (2015). Utility of whole-genome sequencing of *Escherichia coli* O157 for outbreak detection and epidemiological surveillance. J. Clin. Microbiol..

[100] Parsons B.D., Zelyas N., Berenger B.M., Chui L. (2016). Detection, characterization, and typing of Shiga toxin-producing *Escherichia coli*. Front. Microbiol..

