# Supplementary material for: Characterization of Non-O157 Escherichia coli from Cattle Faecal Samples in the North-West Province of South Africa
Source: Microorganisms. 2019 Aug 20;7(8):272. doi: 10.3390/microorganisms7080272 (PMC6723556; doi:10.3390/microorganisms7080272)
Supplement: Supplementary file 1 [file microorganisms-07-00272-s001.pdf]

Table S1: PCR primer sequences.

Gene	Primer sequence (5′ to 3′)	Gene target	Product size (bp)	Reference
ehlyA-F	(F) GCATCATCAAGCGTACGTTCC<br>(R) AATGAGCCAAGCTGGTTAAGCT	*hlyA*	534	(Paton and Paton, 1998)
eaeA	(F) GACCCGGCACAAGCATAAGC<br>(R) CCACCTGCAGCAACAAGAGG	*eaeA*	384	
*stx*1	(F) ATAAATCGCCATTCGTTGACTAC<br>(R) AGAACGCCCACTGAGATCATC	*stx*1	180	
*stx*2	(F) GGCACTGTCTGAAACTGCTCC<br>(R) TCGCCAGTTATCTGACATTCTG	*stx*2	255	
*stx*2a	(F) AGATATCGACCCCTCTTGAA<br>(R) GTCAACCTTCACTGTAAATG	*Stx*2a	969	(He et al., 2012)
uidA	(F) CTGGTATCAGCGCGAAGTCT<br>(R) AGCGGGTAGATATCACACTC	*uidA*	600	(Anbazhagan et al., 2010)
*rfb*O157	(F) CGGACATCCATGTGATATGG<br>(R) TTGCCTATGTACAGCTAATCC	*rfbO157*	259	(Morin et al., 2004)
Streptomycin	(F) TATCCAGCTAAGCGCGAACT<br>(R) ATTTGCCGACTACCTTGGTC	*aadA1*	447	(Momtaz et al., 2012)
Chloramphenicol	(F) AGTTGCTCAATGTACCTATAACC<br>(R) TTGTAATTCATTAAGCATTCTGCC	*catA1*	547	(Van et al., 2008)
Tetracycline A	(F) GGTTCACTCGAACGACGTCA<br>(R) CTGTCCGACAAGTTGCATGA	*tet*A	577	(Randall et al., 2004)
Tetracycline B	(F) CCTCAGCTTCTCAACGCGTG<br>(R) GCACCTTGCTGATGACTCTT	*tet*B	634	
Beta-lactams	(F) ATGAGTATTCAACATTTCCGTGTC<br>(R) TTACCAATGCTTAATCAGTGAGG	*blaTEM-1*	861	(Haidinger et al., 2003)
Biofilm-related	(F) ACTCTGACTTGACTATTACC<br>(R) AGATGCAGTCTGGTCAAC	*csgA*	200	(Knobl et al., 2012)
Biofilm-related	(F) TTTCGATTGTCTGGCTGTATG<br>(R) CTTCAGATTCAGCGTCGTC	*crl*	250	
Biofilm-related	(F) TTATCGCCTGAGGTTATCGTTTGC<br>(R) TCTTCAGGCTCTATTATTCTTCTGGATAT	*csgD*	501	(Ogasawara et al., 2010)
Biofilm-related	(F) TATCGCCTGGATTACTGGCAAC<br>(R) TAGGACGCTGACGTGTCTTATC	*rpoS*	1900	(Olsen et al., 1993)
Biofilm-related	(F) TGCAGAACGGATAAGCCGTGG<br>(R) GCAGTCACCTGCCCTCCGGA	*fimH*	506	(Johnson and Stell, 2000)
Biofilm-related	(F) CCGGCGGGCAATGGGTACA<br>(R) CAGCTCTCACAATCTGGCGAC	*flu*	385	(Restieri et al., 2007)
Biofilm-related	(F) ATATCGGCTAAAGTGGAACAGGTCC<br>(R) TGCTTCCTGGCATTAACTTCACC	*ehaA*[a]	627	(Friedrich et al., 2002)
Biofilm-related	(F) AGTGCATGACTACTGATTGTGCTG<br>(R) CACATTAAACAAACCGCTCTGG	*ehaA*[b]	887	

Table S2: Reference values used to classify isolates as susceptible, intermediate resistant and resistant.

Antimicrobial agent	Disk content	S	I	R
Ampicillin	10 µg	≥ 17	14-16	≤ 13
Amoxicillin-clavulanate	20/10 µg	≥ 18	14-17	≤ 13
Ampicillin-sulbactam	10/10 µg	≥ 15	12-14	≤ 11
Cefepime	30 µg	≥ 25	19-24	≤ 18
Cefotaxime	30 µg	≥ 26	23-25	≤ 22
Cefoxitin	30 µg	≥ 18	15-17	≤ 14
Ceftazidime	30 µg	≥ 21	18-20	≤ 17
Aztreonam	30 µg	≥ 21	18-20	≤ 17
Imipenem	10 µg	≥ 23	20-22	≤ 19
Meropenem	10 µg	≥ 23	20-22	≤ 19
Gentamicin	10 µg	≥ 15	13-14	≤ 12
Streptomycin	10 µg	≥ 15	12-14	≤ 11
Tetracycline	30 µg	≥ 15	12-14	≤ 11
Nalidixic acid	30 µg	≥ 19	14-18	≤ 16
Norfloxacin	10 µg	≥ 17	13-16	≤ 12
Trimethoprim-sulfamethoxazole	1.25/23.75µg	≥ 16	11-15	≤ 10
Chloramphenicol	30 µg	≥ 18	13-17	≤ 12
Colistin	10 µg	≥ 17	13-17	≤ 12

S= Susceptible, I= Intermediate and R= Resistance.

Table S3: Non-O157 *E. coli* isolates sequenced in this study.

Isolate ID	Serogroup	Number of contigs	Total length (bp)	GC (%)	N50
3	O99:H9	116	4686813	50.79	222406
11	O156:H25	299	5181977	50.43	94802
12	O108:H2	103	4881051	50.72	186185
14	O136:H30	128	4564407	50.92	105160
15	O99:H9	103	4686475	50.78	260468
22	wzx-Onovel24:H20	94	4964550	50.53	478048
25	O140:H21	247	5507075	50.47	148987
30	O102:H4	376	5244869	50.65	87095
32	O129:H23	203	5501418	50.53	240397
37	O17:H18	139	5169910	50.45	186852
38	O76:H34	219	4812816	50.88	114298
42	O26:H11	494	5526211	50.4	101076
50	O129:H23	204	5502410	50.52	211833
56	O154:H10	144	4706567	50.66	96083
60	O116:H21	195	5081941	50.7	177288
64	wzx-Onovel5:H19	137	5056230	50.74	161129
65	wzx-Onovel5:H19	135	5058237	50.74	236278
67	O87:H7	127	4764651	50.63	195836
68	O129:H21	171	5105006	50.67	156897
69	O26:H11	478	5562801	50.4	112464
72	O26:H11	107	4610054	50.96	161578
76	O163:H19	303	5171039	50.57	117460
77	O40:H19	156	4930994	50.7	151142
80	O22:H21	87	4795733	50.72	185546

Table S4: Annotation of virulence factors for non-O157 *E. coli* isolates.

Isolate ID	Serogroup	Virulence factor related genes																			
		ehaA	*ehaB*	*epeA*	*agn43*	*espP*	*hlyA*	*subA*	*east1*	*cdtA*	*espA*	*chuA*	*fimH*	*aslA*	*ibeB*	*ompA*	*toxB*	*paa*	*ecpA*	*csgA*	*eaeA*
3	O99:H9	-	+	-	-	-	-	-	-	-	-	-	+	+	+	-	-	-	+	+	-
11	O156:H25	+	+	-	+	+	+	-	+	-	+	-	+	-	+	-	-	+	+	+	+
12	O108:H2	+	+	-	+	-	-	-	-	-	-	-	+	-	+	-	-	-	+	+	-
14	O136:H30	-	+	-	-	-	-	-	-	-	-	-	+	-	+	-	-	-	-	+	-
15	O99:H9	-	+	-	-	-	-	-	-	-	-	-	+	+	+	-	-	-	+	+	-
22	wzx-Onovel24:H20	-	+	-	-	-	-	-	-	-	-	+	+	+	+	-	-	-	+	+	-
25	O140:H21	+	-	-	-	-	-	-	-	-	-	-	+	-	+	-	-	-	+	+	-
30	O102:H4	-	+	-	-	-	-	-	-	-	-	-	+	-	+	-	-	-	.	+	-
32	O129:H23	-	+	-	-	-	-	-	-	-	-	+	+	+	+	-	-	-	+	+	-
37	O17:H18	-	+	-	+	+	+	+	-	-	-	+	+	+	+	+	-	-	+	+	-
38	O76:H34	-	+	-	-	-	-	-	-	-	-	-	+	+	+	-	-	-	+	+	-
42	O26:H11	+	+	-	+	+	+	-	+	-	+	-	+	-	+	-	+	+	+	+	+
50	O129:H23	-	+	-	-	-	-	-	-	-	-	+	+	+	+	-	-	-	+	+	-
56	O154:H10	-	+	-	-	-	-	-	-	-	-	-	+	-	+	-	-	-	+	+	-
60	O116:H21	+	+	+	-	+	+	+	-	-	-	-	+	-	+	-	-	-	+	+	-
64	wzx-Onovel5:H19	-	+	+	+	+	+	+	-	-	-	-	+	-	+	-	-	-	+	+	-
65	wzx-Onovel5:H19	-	+	+	+	+	+	+	-	-	-	-	+	-	+	-	-	-	+	+	-
67	O87:H7	+	+	-	-	-	-	-	-	-	-	-	+	-	+	-	-	-	+	+	-
68	O129:H21	+	+	+	-	+	+	+	-	-	-	-	+	-	+	-	-	-	+	+	-
69	O26:H11	+	+	-	+	+	+	-	+	-	+	-	+	-	+	+	+	+	+	+	+
72	O26:H11	-	+	-	-	-	-	-	-	-	+	-	+	+	+	-	-	-	+	+	-
76	O163:H19	+	+	+	-	-	-	-	-	+	-	-	+	-	+	-	-	-	+	+	-
77	O40:H19	+	+	-	-	+	+	+	-	-	-	-	+	-	+	-	-	-	+	+	-
80	O22:H21	+	+	-	-	+	-	-	-	-	-	-	+	-	+	-	-	-	+	+	-

+ = gene present, - = gene absent

Autotransporter protein genes= (*ehaA*, *ehaB*, *epeA* and *agn43*), serine protease gene= (*espP*), toxins (*hlyA*, *subA*, *east1* and *cdtA*), type III translocated gene (*espA*), iron uptake gene (*chuA*), invasins genes (*fimH*, *aslA*, *ibeB* and *ompA*) and adhesins genes= (*toxB*, *eaeA*, *ecpA*, *paa* and *csgA*).

Table S5: Annotation results of antimicrobial resistance for non-O157 *E. coli*.

**Isolate ID**	**Serogroup**	**Antimicrobial related genes**															
		TEM-105	*TEM-150*	*AadA1*	*aadA2*	*aadA5*	*sul2*	*sul3*	*tet(A)*	*tet(C)*	*tet(D)*	*cmlA6*	*dfrA12*	*linG*	*floR*	*ugd*	*vgaC*
3	O99:H9	-	-	-	-	-	-	-	-	-	-	-	-	-	-	+	-
11	O156:H25	-	-	-	-	-	-	-	-	-	-	-	-	-	-	+	-
12	O108:H2	-	-	-	-	-	-	-	-	-	-	-	-	-	-	+	-
14	O136:H30	-	-	-	-	-	-	-	-	-	-	-	-	-	-	+	-
15	O99:H9	-	-	-	-	-	-	-	-	-	-	-	-	-	-	+	-
22	wzx-Onovel24:H20	-	-	-	-	-	-	-	-	-	-	-	-	-	-	+	-
25	O140:H21	-	-	-	-	-	-	-	-	-	-	-	-	-	-	+	-
30	O102:H4	-	-	-	-	-	-	-	-	-	-	-	-	-	-	+	+
32	O129:H23	-	-	-	-	-	-	-	-	-	-	-	-	-	-	+	-
37	O17:H18	-	-	-	-	-	-	-	-	-	-	-	-	-	-	+	-
38	O76:H34	-	+	-	-	-	+	-	-	+	-	-	-	-	+	+	+
42	O26:H11	-	-	-	-	-	+	-	+	-	+	-	-	-	-	+	+
50	O129:H23	-	-	-	-	-	-	-	-	-	-	-	-	-	-	+	-
56	O154:H10	+	-	+	+	-	-	+	+	-	+	+	+	+	-	+	-
60	O116:H21	-	-	-	-	-	-	-	-	-	-	-	-	-	-	-	-
64	wzx-Onovel5:H19	-	-	-	-	-	-	-	-	-	-	-	-	-	-	-	-
65	wzx-Onovel5:H19	-	-	-	-	-	-	-	-	-	-	-	-	-	-	-	-
67	O87:H7	-	-	-	-	-	-	-	-	-	-	-	-	-	-	-	-
68	O129:H21	-	-	-	-	-	-	-	-	-	-	-	-	-	-	-	-
69	O26:H11	-	-	-	-	-	+	-	+	-	+	-	-	-	-	+	+
72	O26:H11	-	-	-	-	-	-	-	-	-	-	-	-	-	-	+	-
76	O163:H19	-	-	-	-	-	-	-	-	-	-	-	-	-	-	+	-
77	O40:H19	-	-	-	-	-	-	-	-	-	-	-	-	-	-	+	-
80	O22:H21	-	-	-	-	+	+	-	-	+	-	-	-	-	-	+	-

+ = gene present, - = gene absent

Beta-lactams (*TEM-105* and *TEM-150*), streptomycin (*aadA1*, *aadA2 aadA5*), sulfonamide (*sul2* and *sul3*), tetracycline (*tetACD*), phenicol (*cmlA6* and *floR*), diaminopyrimidine (*dfrA12*), lincosamides (*linG*), polymyxin (*ugd*) and streptogramin (*vgaC*).

Table S6: Annotated results of plasmids search for non-O157 *E. coli*.

Isolate ID	Serogroup	Plasmids types															
		Col156_1	ColRNAI_1	ColpVC_1	IncB/O/K/Z_2	IncB/O/K/Z_3	IncB/O/K/Z_4	IncFIA_1	IncFIB(AP001918)_1	IncFIB(K)_1_Kpn3	IncFIC(FII)_1	IncFII(pHN7A8)_1_pHN7A8	IncHI2_1	IncI1_1_Alpha	IncI2_1_Delta	IncR_1	IncY_1
3	O99:H9	-	-	+	-	-	-	-	-	-	-	-	-	-	-	-	-
11	O156:H25	+	-	-	-	-	-	-	+	-	-	-	-	-	-	-	-
12	O108:H2	-	-	-	-	-	-	-	-	-	-	-	-	-	-	-	-
14	O136:H30	-	+	-	-	-	-	-	-	-	-	-	-	-	-	-	-
15	O99:H9	-	-	+	-	-	-	-	-	-	-	-	-	-	-	-	-
22	wzx-Onovel24:H20	-	.	-	-	-	-	+	+	-	+	-	-	-	+	-	-
25	O140:H21	-	-	-	-	-	-	+	+	-	+	-	-	-	-	-	-
30	O102:H4	-	-	-	-	-	-	-	+	-	+	-	-	+	-	-	-
32	O129:H23	-	+	-	-	-	-	-	+	-	-	-	-	+	-	-	-
37	O17:H18	-	-	-	-	-	-	-	+	-	+	-	-	-	-	-	-
38	O76:H34	-	+	-	-	-	-	-	-	+	-	-	+	-	-	+	-
42	O26:H11	-	+	-	-	+	-	-	+	-	-	-	-	-	-	-	-
50	O129:H23	-	+	-	-	-	-	-	+	-	-	-	-	+	-	-	-
56	O154:H10	-	-	-	-	-	-	-	-	-	-	-	-	-	-	-	+
60	O116:H21	-	+	-	+	-	-	-	+	-	-	+	-	-	-	-	-
64	wzx-Onovel5:H19	-	+	-	+	-	-	-	+	-	-	-	-	-	-	-	-
65	wzx-Onovel5:H19	-	+	-	+	-	-	-	+	-	-	-	-	-	-	-	-
67	O87:H7	-	-	-	-	-	-	+	+	-	+	-	-	-	-	-	-
68	O129:H21	-	+	-	+	-	-	-	+	-	-	+	-	-	-	-	-
69	O26:H11	-	+	-	-	+	-	-	+	-	-	-	-	-	-	-	-
72	O26:H11	-	+	-	-	-	-	-	-	-	-	-	-	-	-	-	-
76	O163:H19	-	-	-	-	-	+	-	-	-	-	-	-	-	-	-	-
77	O40:H19	-	+	-	-	-	-	+	+	-	+	-	-	-	-	-	-
80	O22:H21	-	-	-	-	-	-	-	-	-	-	-	-	-	-	-	+

+ = gene present, - = gene absent.

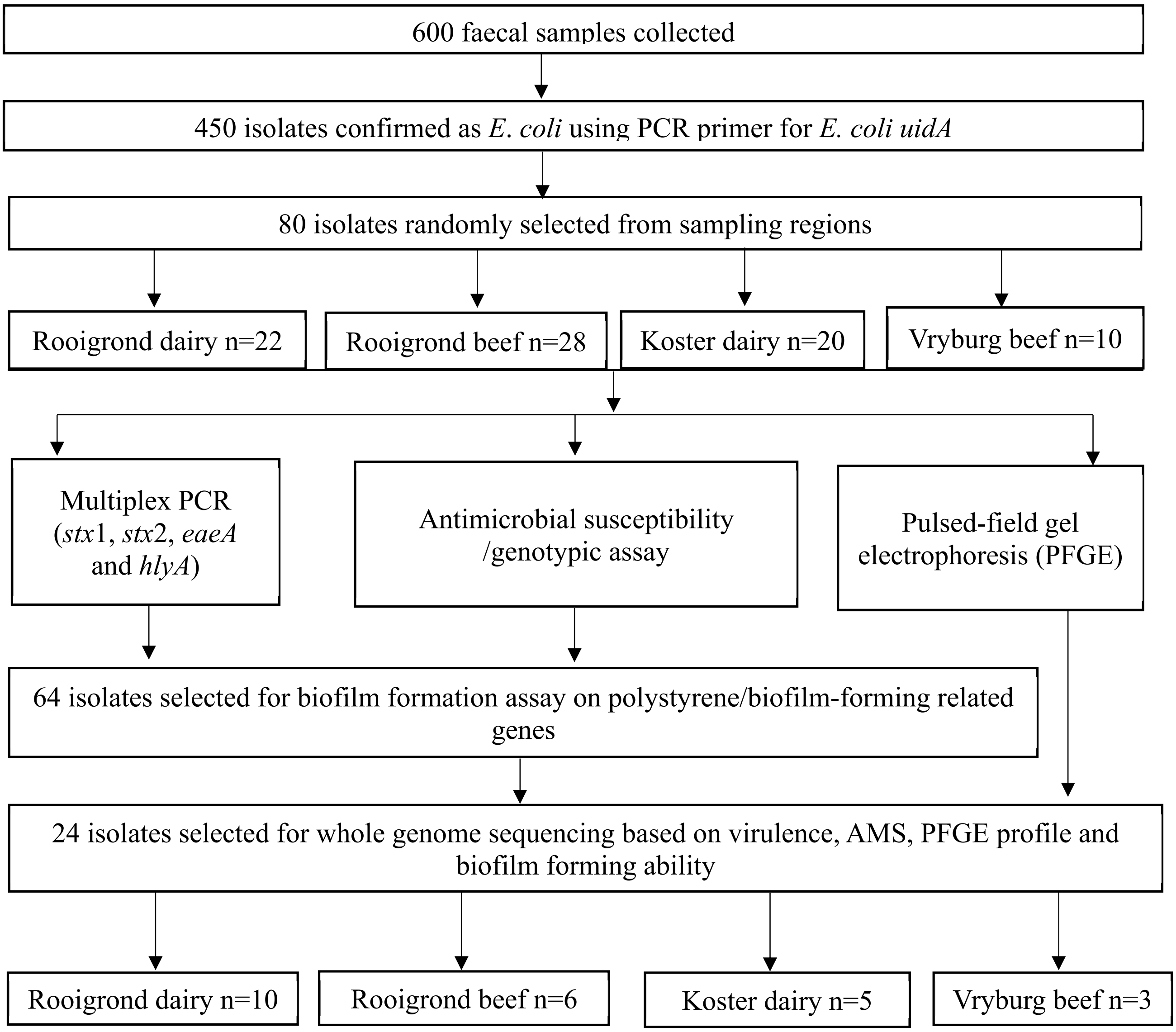

Figure S1: A flow chart illustrating bacterial characterization.

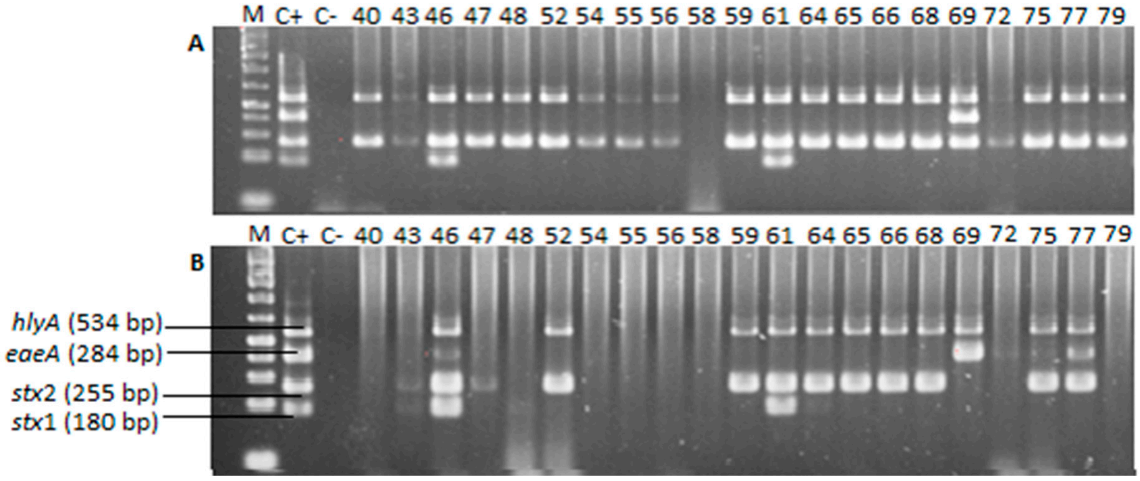

Figure S2: Multiplex gel image of amplicons indicating no detection of *stx*2 (gel B). M indicates molecular marker 1kb plus, C+ the positive control, C- the negative control and isolate number (40-79).
